# NKD1 enhances colon cancer progression by inhibiting the autophagic degradation of MYC

**DOI:** 10.1038/s41419-025-07875-8

**Published:** 2025-07-17

**Authors:** Wenbin Lu, Jianjun Tang, Yue Wang, Xiaoyan Gu, Hua Zhang, Yungang Liu, Ying Xiao, Qi Zhu, Jianzhong Deng, Ying Shen, Anqi Jiang, Yixin Xu, Jianhua Jin, Yongzhong Hou, Qian Liu

**Affiliations:** 1 Department of Oncology, The Wujin Clinical College of Xuzhou Medical University, Changzhou, Jiangsu 213017 China; 2 Department of Oncology, Wujin Hospital Affiliated with Jiangsu University, Changzhou, Jiangsu 213017 China; 3 Changzhou Key Laboratory of Molecular Diagnostics and Precision Cancer Medicine/Wujin Institute of Molecular Diagnostics and Precision Cancer Medicine of Jiangsu University, Changzhou, Jiangsu 213017 China; 4 Cancer Institute, Xuzhou Medical University, Xuzhou, Jiangsu 221004 China

**Keywords:** Colon cancer, Cell growth, Oncogene proteins

## Abstract

NKD1 is a known suppressor of the Wnt/β-catenin pathway. However, our previous study revealed that NKD1 could promote the proliferation and migration of colon cancer cells, enhancing colon cancer progression via unknown mechanisms. In the present study, we found that peroxisome proliferator-activated receptor δ (PPARδ) is a transcription factor of the NKD1 gene. By analyzing the differential protein expression profiles between SW620 and SW620-nkd1^−/−^ cells, we found that NKD1 dramatically increased MYC protein expression. Further study revealed that the MYC protein was degraded mainly through the autophagy pathway in colon cancer cells and that NKD1 restrained this process by suppressing the interaction between LC3B and MYC proteins. Interestingly, we found that NKD1 inhibited the autophagy signaling pathway. In-depth research revealed that NKD1 bound to the MYC protein through the EF-hand domain, facilitating the entry of the MYC protein into the nucleus and inhibiting cell apoptosis. Moreover, NKD1 activated the expression of MYC downstream target genes through MYC. Functionally, the PPARδ/NKD1/MYC signaling pathway increased colon cancer cells’ proliferation, migration and angiogenesis capabilities. Therefore, NKD1 may serve as a specific biomarker for colon cancer and a potential new target for tumor treatment.

## Introduction

Naked cuticle (nkd) was first identified in *Drosophila*, and *Drosophila* Naked Cuticle (Nkd) functions as an antagonist of the canonical Wnt signaling pathway [1]. Mouse Nkd is a Dishevelled binding protein that functions as a negative regulator of the Wnt/β-catenin pathway [2]. In zebrafish blastula cells, NKD1 is recruited to the Wnt signalosome with Dvl2, which was activated to interact with β-catenin, suppressing its nuclear entry [3]. The human NKD protein has two homologs, NKD1 and NKD2. Researchers have reported that NKD1 is expressed at low levels in breast cancer tissues, hepatocellular carcinoma, and acute myeloid leukemia [4–6]; However, it is also highly expressed in colorectal, gastric and pancreatic cancer cells [2]. Moreover, our previous studies indicated that NKD1 is highly expressed in colorectal carcinoma tissues and promotes the proliferation and migration of colon cancer cells [7, 8]. However, the underlying mechanism by which NKD1 enhances the progression of colon cancer remains obscure.

PPARδ (peroxisome proliferator-activated receptor δ) is a member of the PPAR family and is a ligand-activated transcription factor. PPARs are activated by binding with corresponding ligands. Activated PPARs form heterodimers with RXRs, which combine with promoter sequences of specific genes to regulate gene expression [9]. Studies have shown that PPARδ promotes the proliferation and migration of colon cancer cells [10, 11]. However, the mechanism by which PPARδ promotes the progression of colon cancer remains unclear.

c-MYC is a widely studied oncoprotein that promotes tumor proliferation and migration by regulating the expression of numerous downstream target genes [12, 13]. Studies have demonstrated that the c-MYC protein is degraded through the proteasome pathway in most tumor cells [14–17]; however, its degradation pathway in colon cancer cells has not yet been reported.

In this study, we investigated the upstream regulatory mechanism of NKD1 and revealed that PPARδ is a transcription factor of the NKD1 gene. PPARδ promoted the proliferation and migration of colon cancer cells by increasing NKD1 transcription. We analyzed the differential protein expression profiles between parental SW620 cells and SW620-nkd1^−/−^ cells, in which the NKD1 gene was knocked out in SW620 cells. Research has shown that NKD1 knockout significantly reduces the expression of the MYC protein and that there is a significant positive correlation between the expression of NKD1 and that of MYC in colon cancer tissues. Further studies revealed that in colon cancer cells, the MYC protein is degraded through the autophagy pathway and that NKD1 hampers this process by suppressing the interaction between the MYC and LC3B proteins. Moreover, NKD1 bound to the MYC protein through the EF-hand domain, facilitating the entry of the MYC protein into the nucleus and suppressing cell apoptosis. Functionally, the PPARδ/NKD1/MYC signaling pathway increased colon cancer cells’ proliferation, migration and angiogenesis capabilities in *vitro* and in *vivo*, suggesting that NKD1 could serve as a potential therapeutic target for colon cancer diagnosis and treatment.

## Results

### PPARδ is a transcription factor of the NKD1 gene

**Fig. 1:**
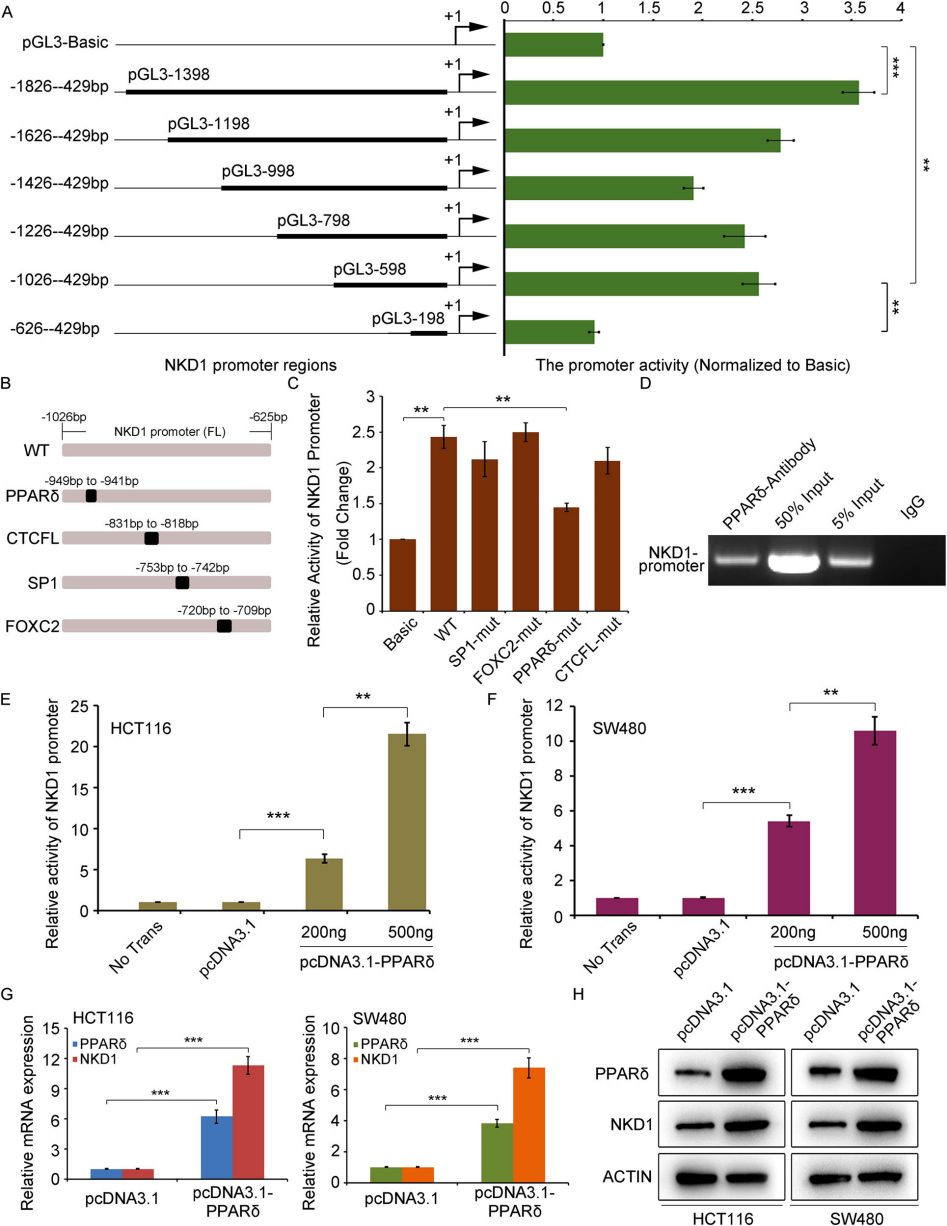
PPARδ is a transcription factor of the NKD1 gene. **A** Relative activities of different truncated promoter regions of NKD1 gene were detected by Dual Luciferase system in HCT116 cells transfected transiently with different NKD1 promoter fragments (100 ng), and pRL-TK plasmids (50 ng), respectively. **B** The pattern diagram of potential transcription factor binding sites in the promoter sequence of NKD1 gene (−1026 to −625 bp). **C** The promoter activities of deletion mutations of different transcription factor binding motif in NKD1 promoter regions (−1026 to −429 bp) were detected by dual luciferase reporter assays. **D** The ability of PPARδ protein binding to NKD1 promoter was detected by Chromatin immunoprecipitation (ChIP) experiments with colon cancer HCT116 cells. **E** Relative activities of NKD1 promoter were detected by Dual luciferase system in colon cancer HCT116 or SW480 (**F**) cells transiently transfected with pcDNA3.1 plasmids (500 ng) or pcDNA3.1-PPARδ (200 ng or 500 ng), respectively. **G** The mRNA or protein (**H**) expression levels of NKD1 in HCT116 cells or SW480 cells transiently transfected with pcDNA3.1 plasmids (500 ng) or pcDNA3.1-PPARδ (500 ng).

The upstream regulatory mechanism of the NKD1 gene is unclear. To investigate the transcription factors that regulate the NKD1 gene, we individually inserted the NKD1 promoter sequence (−1826 to −429 bp) and the truncated promoter fragments into the dual-luciferase reporter vector pGL3-Basic. The dual-luciferase reporter results revealed that different truncated promoter fragments exhibited different activities (Fig. 1A), among which the pGL3-1198, pGL3-998, pGL3-798, and pGL3-598 promoter regions presented similar promoter activities. However, the promoter activity of the pGL3-198 fragment suddenly decreased to the basic plasmid level (negative control) level, indicating that the potential transcription factors of the NKD1 gene bound to the promoter region (−1026 to −626 bp). The promoter sequence (−1026 to −626 bp) was analyzed via the transcription factor online prediction website (http://alggen.lsi.upc.es/cgi-bin/promo_v3/promo/promoinit.cgi?dirDB=TF_8.3), and the prospective binding motifs for the SP1, FOXC2, PPARδ, and CTCFL transcription factors according to the website score were determined. We then created deletion mutations in the binding motifs (Fig. 1B). The dual-luciferase reporter results revealed that only knockout mutations in the PPARδ binding motif resulted in a significant decrease in promoter activity (Fig. 1C), indicating that the transcription factor PPARδ enhances NKD1 promoter activity. Chromatin immunoprecipitation (ChIP) revealed that PPARδ proteins bind to the NKD1 promoter (Fig. 1D), further confirming that PPARδ is a transcription factor of the NKD1 gene. To investigate whether PPARδ regulates the activity of the NKD1 promoter, we transfected the pGL3-598 promoter plasmid, pcDNA3.1 plasmid, and pcDNA3.1-PPARδ plasmid into colon cancer HCT116 and SW480 cells, and the NKD1 promoter activity regulated by PPARδ was detected via dual-luciferase reporter gene analysis. The results revealed that PPARδ dose-dependently increased the promoter activity of the NKD1 gene (Fig. 1E, F). Moreover, the pcDNA3.1 and pcDNA3.1-PPARδ plasmids were transfected into colon cancer HCT116 and SW480 cells, respectively. qRT-PCR and Western blot assays revealed that the overexpression of PPARδ considerably increased the expression levels of NKD1 mRNA and protein (Fig. 1G, H). These findings indicate that PPARδ is a transcription factor of the NKD1 gene in colon cancer cells.

### PPARδ promotes the proliferation and migration of colon cancer cells through NKD1

**Fig. 2:**
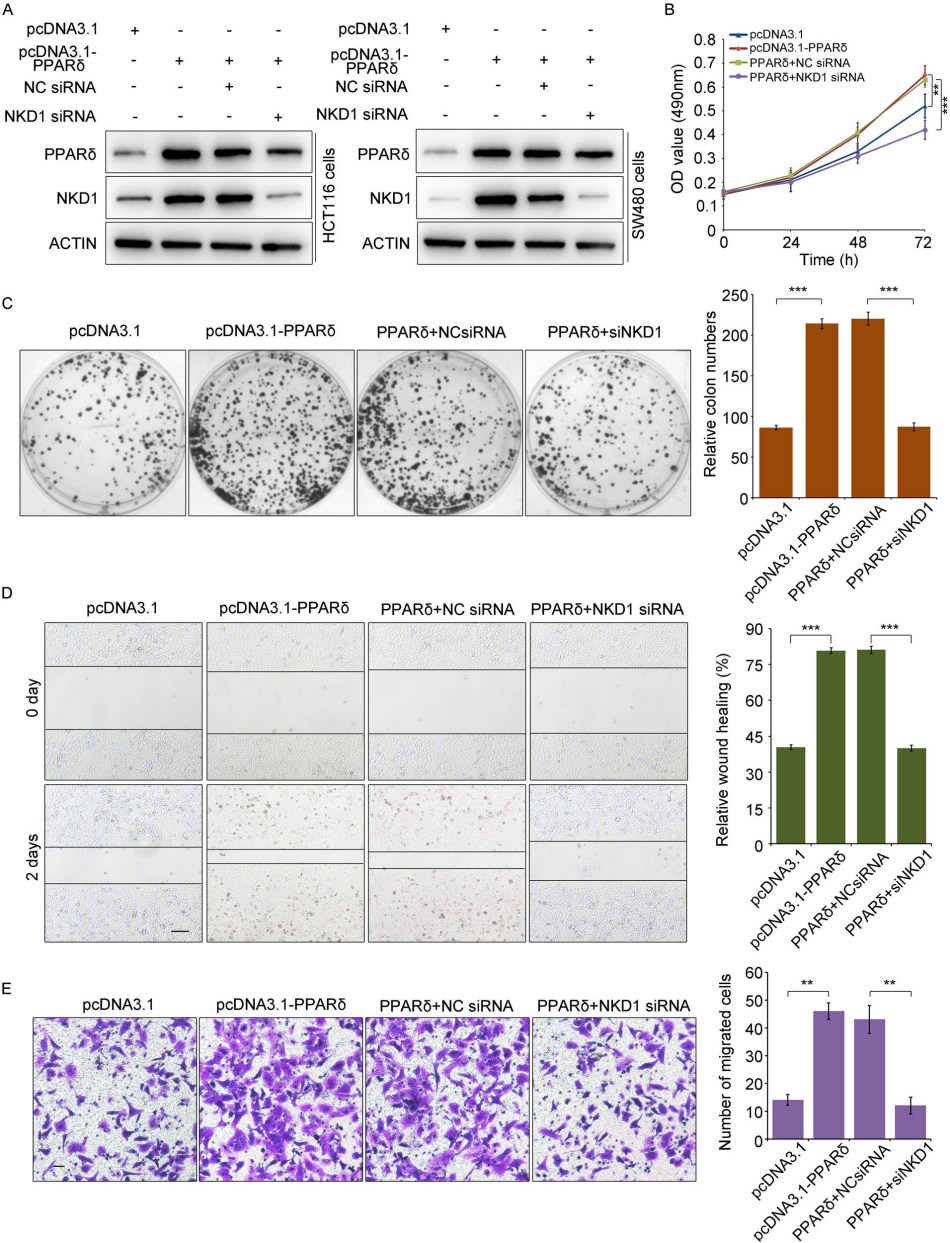
PPARδ activates colon cancer cell proliferation and migration through NKD1. **A** The protein levels of NKD1 and PPARδ were detected by western blotting in the HCT116 cells and SW480 cells transfected transiently with pcDNA3.1 plasmids (500 ng), pcDNA3.1-PPARδ (500 ng), Negative Control (NC) siRNA (100 nM) or NKD1 siRNA (100 nM), as shown in the figure. **B** Cell growth abilities were detected through MTT assays and Clone formation experiments (**C**) in colon cancer HCT116 cells transfected with pcDNA3.1 plasmids (500 ng), pcDNA3.1-PPARδ (500 ng), pcDNA3.1-PPARδ (500 ng) + NC siRNA (100 nM), and pcDNA3.1-PPARδ (500 ng) + NKD1 siRNA (100 nM), respectively. The bar chart on the right showed the numbers of clones under different statistical conditions. **D** The cell migration abilities were detected through Scratch experiments and Transwell experiments (**E**) in colon cancer HCT116 cells transfected with pcDNA3.1 plasmids (500 ng), pcDNA3.1-PPARδ (500 ng), pcDNA3.1-PPARδ (500 ng) + NC siRNA (100 nM), and pcDNA3.1-PPARδ (500 ng) + NKD1 siRNA (100 nM), respectively. The bar chart on the right were the statistics of different wound healing and the number of migrated cells. Each experiments were repeated at least three times, *p <0.05, **p <0.01, ***p <0.001.

We found that PPARδ is a transcription factor of the NKD1 gene, and our previous studies demonstrated that NKD1 promotes colon cancer cell proliferation and migration [7, 8]. We next investigated whether PPARδ could promote colon cancer cell proliferation and migration by activating NKD1 transcription. The pcDNA3.1 plasmid, pcDNA3.1-PPARδ plasmid, pcDNA3.1-PPARδ + negative control siRNA (NC siRNA), and pcDNA3.1-PPARδ + NKD1 siRNA were consecutively transfected into colon cancer HCT116 and SW480 cells. Western blotting demonstrated that the overexpression of PPARδ critically increased the protein expression of NKD1, nevertheless the simultaneous overexpression of PPARδ and the knockdown of the NKD1 gene strongly reduced the protein expression of NKD1 (Fig. 2A). MTT assays and colony formation experiments revealed that the overexpression of PPARδ notably increased the colon cancer cell proliferation and that PPARδ overexpression with NKD1 gene expression knockdown remarkably hindered cell proliferation (Fig. 2B, C), indicating that PPARδ promotes colon cancer cell proliferation through NKD1. Additionally, scratch and Transwell assays indicated that the overexpression of PPARδ greatly increased the migration of colon cancer cells. PPARδ overexpression with NKD1 gene knockdown markedly impeded the migration of colon cancer cells (Fig. 2D, E), indicating that PPARδ enhances colon cancer cell migration *via* NKD1. In conclusion, the above results suggest that PPARδ promotes colon cancer cell proliferation and migration by transcriptionally activating the expression of the NKD1 gene.

### NKD1 promotes MYC protein expression in colon cancer cells

**Fig. 3:**
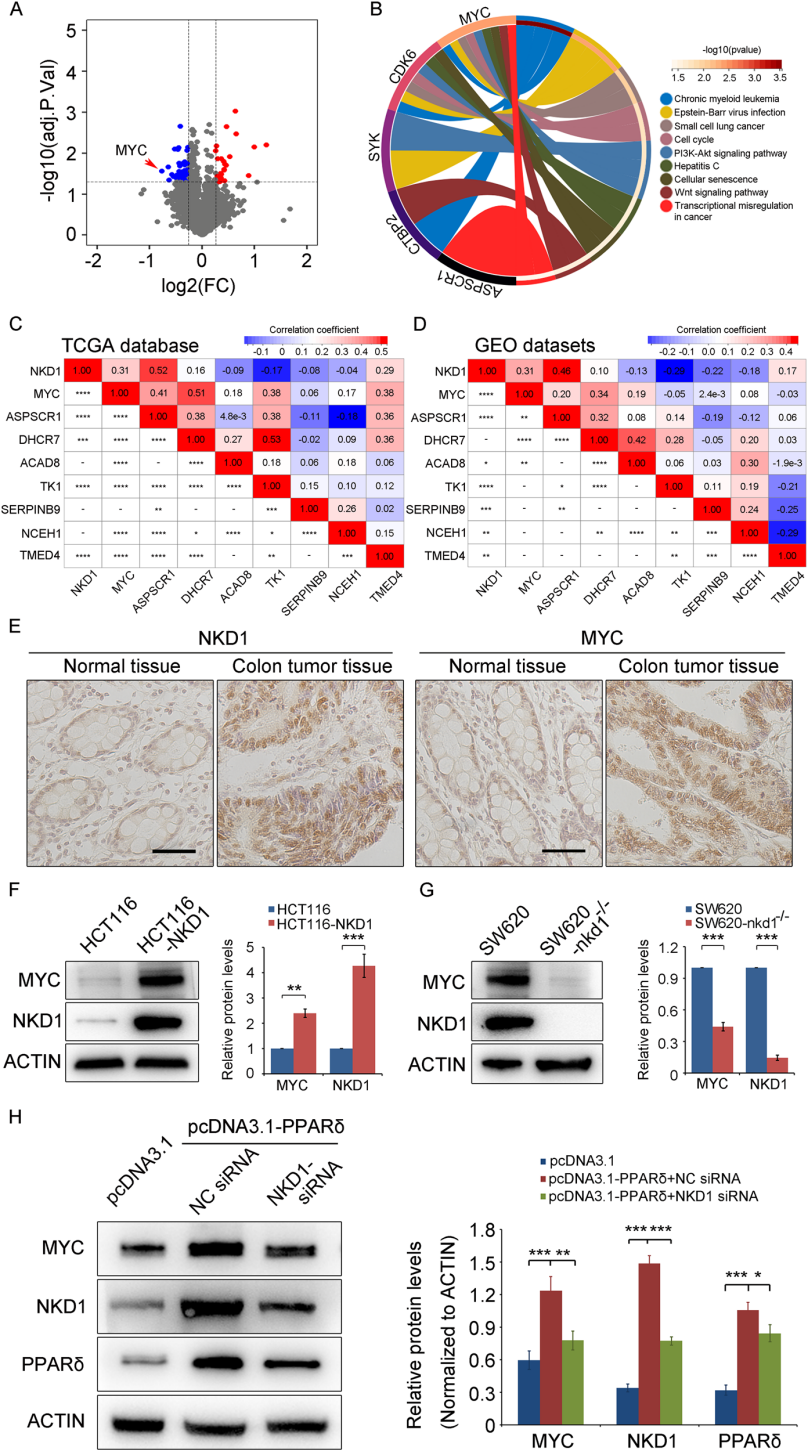
NKD1 upregulates MYC protein expression in colon cancer cells. **A** Volcano plots of differential protein expressions between SW620-nkd1^−/−^ cells and SW620 cells measured by Isotopic Labeling Relative and Absolute Quantitative Techniques. Red dots represented the raised proteins in the SW620-nkd1^−/−^ cells and Blue dots represented the reduced proteins, the Grey dots mean the proteins without notable changes. The screen was executed on the basis of the adjusted *p*-value < 0.05. **B** Chord diagram summarizing KEGG pathway enrichment for five hub genes. The left semicircle lists the hub genes. The right semicircle lists the enriched pathways, each pathway segment colored as in the legend. Ribbons connect each gene to the pathways in which it is involved; ribbon width is proportional to the overlap/enrichment strength, while ribbon color intensity corresponds to –log10(p‑value) (deeper red = higher significance). **C** The correlation between the NKD1 and MYC expression using transcriptome data from TCGA-COAD database were analyzed via the SangerBox 3.0 network software (http://www.sangerbox.com/home.html). **D** Correlation analysis of NKD1 and MYC gene transcriptome data downloaded from GEO datasets (GSE17536 and GSE29621) was conducted with the SangerBox 3.0 network software. **E** The protein expression levels of NKD1 and MYC in normal colon tissues and colon cancer tissues were detected by Immunohistochemistry experiments. The scale bar was 100 μm. **F** Protein levels of MYC and NKD1 were detected by Western blotting in HCT116 and HCT116-NKD1 cells or SW620 and SW620-nkd1^−/−^ cells (**G**). On the right was the quantitative analysis of the grayscale values of protein bands. **H** Protein levels of MYC, NKD1, and PPARδ were determined by western blotting in HCT116 cells transiently transfected with pcDNA3.1 plasmid (500 ng), pcDNA3.1-PPARδ plasmid (500 ng) + negative control (NC) siRNA (100 nM), pcDNA3.1-PPARδ plasmid (500 ng) + NKD1 siRNA (100 nM); on the right were a quantitative analysis of the grayscale values of protein bands. Each experiments were repeated at least three times, *p <0.05, **p <0.01, ***p <0.001.

No signaling pathways have been reported to be associated with NKD1. To investigate the downstream regulatory proteins of NKD1, differential protein expression profiling analysis of SW620 cells and SW620-nkd1^−/−^ cells was conducted with isobaric tags for relative and absolute quantitation technology by Sangon Biotech Company. The results revealed that the expression levels of 49 proteins were markedly reduced in SW620-nkd1^−/−^ cells relative to SW620 cells (*p* < 0.05) (Fig. 3A and Supplementary Table ST2). Knockout of the NKD1 gene resulted in substantial changes in these proteins, which are closely related to the function of the NKD1 protein. Therefore, we performed a Kyoto Encyclopedia of Genes and Genomes (KEGG) analysis of the signaling pathways and diseases associated with the top 20 genes demonstrating reduced protein expression after NKD1 gene knockout. The results revealed that the top 20 genes demonstrating strongly reduced protein expression after NKD1 gene knockout were involved in the PI3K-Akt signaling pathway, cellular senescence and Wnt signaling pathway. These genes were also involved in diseases such as Chronic myeloid leukemia, Small cell lung cancer, Hepatitis carcinoma (Fig. 3B). These results suggest that NKD1 is involved in the Wnt signaling pathway, consistent with existing relevant research reports [3, 7, 8]. We next analyzed the protein sets involved in signaling pathways or diseases, revealing that the MYC protein was involved in most of these (Supplementary Table ST3). Additionally, the correlations between the NKD1 and MYC transcriptome data downloaded from TCGA-COAD database and GEO datasets were analyzed via the Sangerbox 3.0 online software (http://www.sangerbox.com/home.html) (Fig. 3C, D and Supplementary Table ST4). The results revealed a significant positive correlation between NKD1 and MYC gene expression in colon cancer tissues (*p* < 0.05). Moreover, the expression levels of NKD1 and MYC in normal colon tissues and colon cancer tissues were determined via immunohistochemistry (IHC). The results revealed that NKD1 and MYC were expressed at low levels in normal tissue and highly expressed in colon cancer tissues (Fig. 3E). The regulatory effect of NKD1 on the MYC protein in stable transgenic cell lines with the NKD1 gene overexpressed or knocked out was also investigated, revealing that MYC expression was notably greater in HCT116-NKD1 versus HCT116 cells (Fig. 3F). In contrast, SW620-nkd1^−/−^ cells demonstrated markedly lower MYC expression than that in SW620 cells (Fig. 3G), indicating that NKD1 increases MYC protein expression levels in colon cancer cells. Next, we investigated whether PPARδ could regulate the expression of MYC protein through NKD1, the results revealed that PPARδ overexpression obviously increased the protein expression of NKD1 and MYC; overexpression of PPARδ while knocking down NKD1 expression led to a decrease in MYC protein expression, indicating that PPARδ upregulates MYC protein expression through NKD1. Overall, the above results demonstrate a positive correlation between NKD1 and MYC expression in colon cancer tissues. Furthermore, NKD1 notably increases MYC protein expression in colon cancer cells.

### NKD1 inhibits autophagic degradation of the MYC protein

**Fig. 4:**
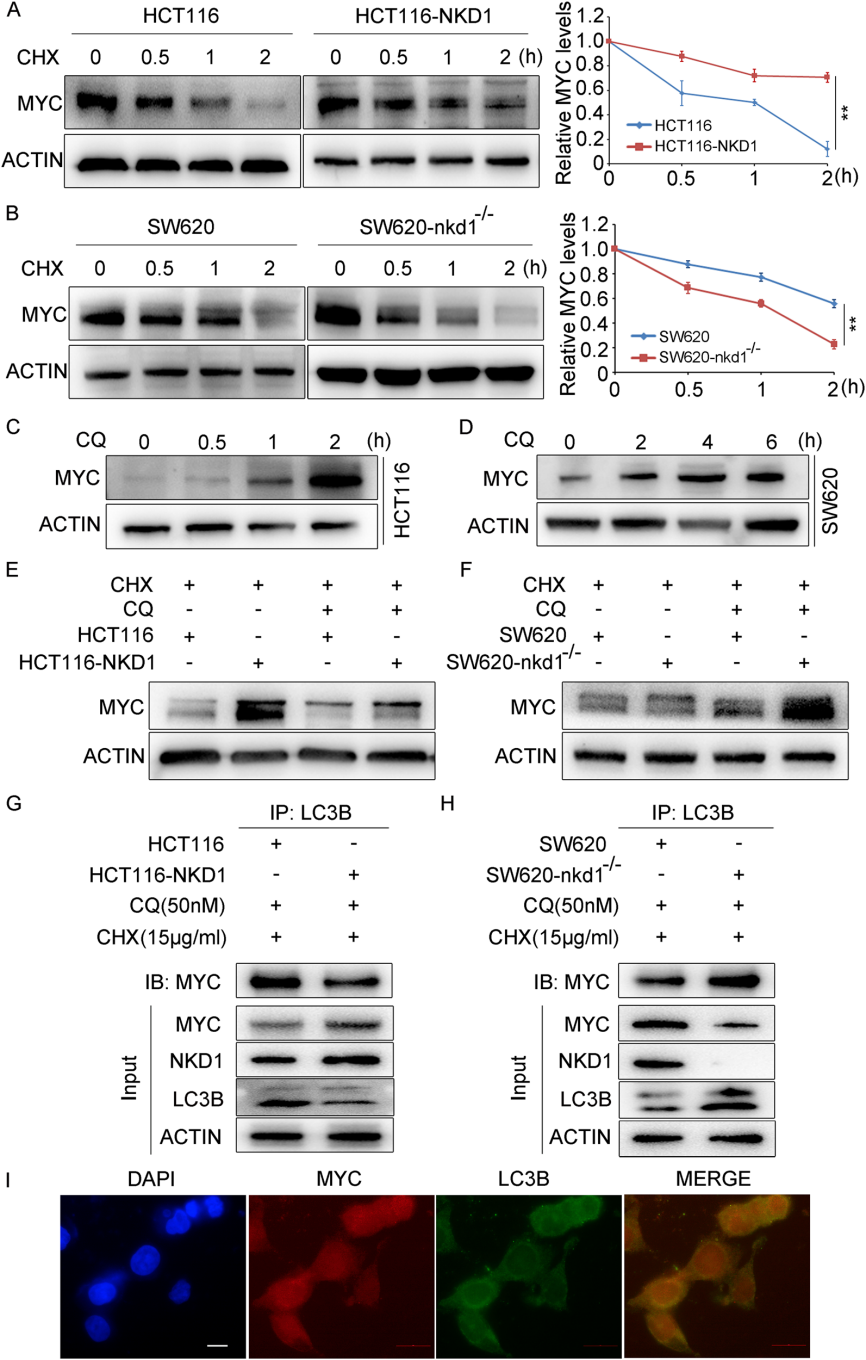
NKD1 inhibits the autophagic degradation of MYC proteins. **A** The protein expressions of MYC genes in parental HCT116 cells and HCT116-NKD1 cells, or in parental SW620 cells and SW620-nkd1^−/−^ cells (**B**) treated with Cycloheximide (CHX, an inhibitor of protein synthesis, 15 μg/ml) for 0, 0.5, 1, and 2 h was disclosed by western blotting, the right result was a quantitative analysis of the grayscale values of protein bands through Image J software. **C** Western blotting measured the protein levels of MYC in colon cancer HCT116 cells or SW620 cells (**D**) treated with Chloroquine (CQ, an autophagy inhibitor, 50 nM) for different time points, respectively. **E** The protein levels of MYC genes in parental HCT116 cells and HCT116-NKD1 cells, or parental SW620 cells and SW620-nkd1^−/−^ cells (**F**) treated with CHX or (and) CQ as indicated by the mark, respectively, were detected by Western blotting. **G** The binding abilities of LC3B (an autophagy marker) and MYC proteins in parental HCT116 cells and HCT116-NKD1 cells, or parental SW620 cells and SW620-nkd1^−/−^ cells (**H**) treated with CQ (50 nM) and CHX (15 μg/ml) were measured by immunoprecipitation assays. **I** The colocalization of MYC and LC3B proteins was measured by the immunofluorescences, the scale bar is 50 μm. Each experiments was repeated at least three times, *p <0.05, **p <0.01, ***p <0.001.

To better understand the molecular mechanisms by which NKD1 regulates MYC protein expression, we first determined the localization of the NKD1 and MYC proteins in colon cancer cells through laser confocal microscopy experiments, demonstrating that the NKD1 proteins were distributed mainly in the cytoplasm, with a small concentration in the nucleus. The MYC protein was distributed in both the nucleus and the cytoplasm. Moreover, there was a clear co-localization between the NKD1 and MYC proteins in the cytoplasm and the nucleus (Supplementary Fig. SF1). These results suggest that NKD1 has played more biological functions in the cytoplasm; therefore, we first analyzed the regulatory effects of NKD1 on the MYC protein at the protein level. HCT116, HCT116-NKD1 cells, SW620, and SW620-nkd1^−/−^ cells were treated with cycloheximide (CHX, a protein synthesis inhibitor) for different durations to determine the effect of NKD1 on the half-life of the MYC protein. The results demonstrated that the half-life of the MYC protein in HCT116-NKD1 cells was markedly greater than that in HCT116 cells (Fig. 4A), whereas the half-life of the MYC protein in SW620-nkd1^−/−^ cells was obviously lower than that in SW620 cells (Fig. 4B), indicating that NKD1 markedly suppresses the degradation of MYC proteins. Many investigations have demonstrated that the MYC protein is degraded through the proteasome ubiquitination pathway [18–20]; however, the degradation mode of the MYC protein in colon cancer cells has not yet been reported. To better understand the degradation mode of the MYC protein in colon cancer cells, colon cancer HCT116 or SW620 cells were treated with MG132 (a proteasome inhibitor) or chloroquine (CQ, an autophagy inhibitor), respectively. Compared with the cells treated with DMSO, the cells treated with CQ notably accumulated the MYC protein within 2 h (Fig. 4C, D), whereas the accumulation rate of the MYC protein in the cells treated with MG132 was much lower (Supplementary Fig. SF2). These results demonstrate that MYC proteins are mainly degraded through the autophagy pathway in colon cancer cells. HCT116 and HCT116-NKD1 cells were treated with CHX to investigate the effect of NKD1 on the autophagic degradation of the MYC protein; one group of cells was treated with CQ, and the other group of cells was not. Western blotting revealed that the protein expression level of MYC in HCT116-NKD1 cells treated with CHX and CQ was fundamentally lower than that in cells treated with CHX but not with CQ (Fig. 4E), indicating that NKD1 overexpression inhibits MYC autophagic degradation. The SW620 and SW620-nkd1^−/−^ cells were also subjected to these experiments. The expression levels of the MYC protein in SW620-nkd1^−/−^ cells treated with CHX and CQ were notably greater than those in the cells treated with only CHX (Fig. 4F), indicating that NKD1 gene knockout considerably increases MYC protein autophagic degradation in colon cancer cells. Next, we investigated how the NKD1 protein blocks this process. LC3B is an important biomarker for autophagic degradation and is responsible for transporting proteins that need to be degraded by autophagy to the autophagosome [21]. Colon cancer HCT116 and HCT116-NKD1 cells, or SW620 and SW620-nkd1^−/−^ cells were treated with CHX and CQ, and LC3B antibodies were used to isolate the LC3B proteins from the cell lysates. Western blotting revealed that the binding ability between the LC3B and MYC proteins in HCT116-NKD1 cells was exceptionally lower than that in HCT116 cells (Fig. 4G), whereas the binding ability between the LC3B and MYC proteins in SW620-nkd1^−/−^ cells was significantly stronger than that in SW620 cells (Fig. 4H), indicating that NKD1 represses MYC protein autophagic degradation by inhibiting the ability of LC3B to bind the MYC protein. Furthermore, immunofluorescence experiments revealed an obvious interaction between MYC and LC3B in colon cancer cells (Fig. 4I). In short, NKD1 could maintain high MYC expression levels in cells by suppressing MYC protein autophagic degradation.

### NKD1 suppresses the autophagic signaling pathway in colon cancer cells

**Fig. 5:**
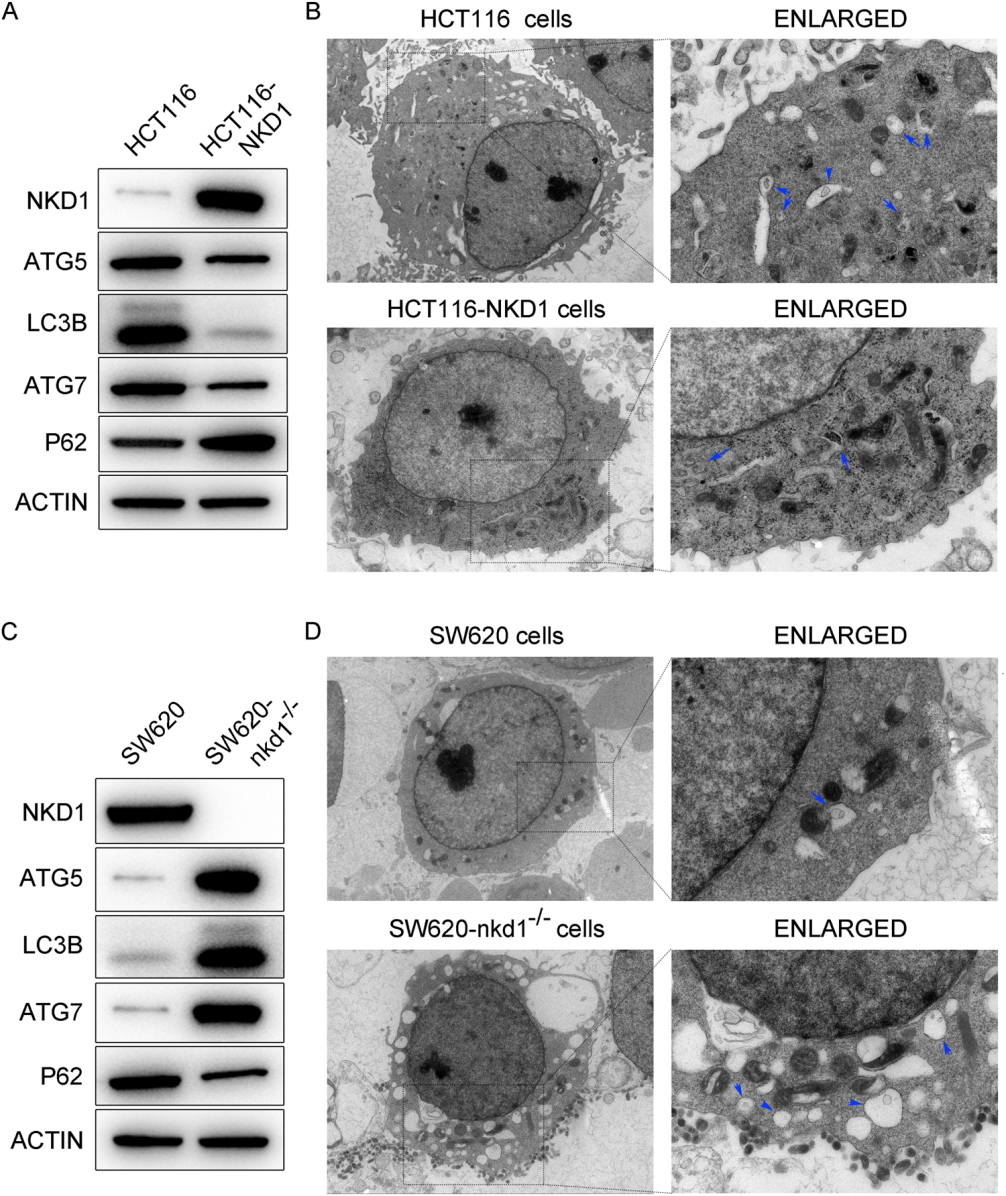
NKD1 inhibits autophagy signaling pathway in colon cancer cells. **A** The protein levels of autophagy pathway marker (ATG5, ATG7, LC3B, and P62) in HCT116 cells and HCT116-NKD1 cells were determined by western blotting. **B** Transmission Electron Microscopy analyzed the autophagosomes in HCT116 and HCT116-NKD1 cells. Single intact cells are photographed with 3.0 K zoom, and local areas are magnified to 10.0 K zoom for photography. **C** The expression levels of autophagy pathway marker protein in SW620 and SW620-nkd1^−/−^ cells were determined by western blotting. **D** Transmission Electron Microscopy analyzed the autophagosomes in SW620 and SW620-nkd1^−/−^ cells. Single intact cells are photographed with 3.0 K zoom, and local areas are magnified to 10.0 K Zoom for photography. Each experiments was repeated at least three times, *p <0.05, **p <0.01, ***p <0.001.

The results of the immunoprecipitation experiments (Fig. 4G, H) revealed that NKD1 could inhibit the expression of the LC3B protein; thus, we investigated whether NKD1 could regulate other autophagy markers. HCT116 cells and HCT116-NKD1 cells were assessed by Western blotting to determine whether NKD1 could regulate the expression levels of autophagy markers (such as ATG5, ATG7 [22], LC3B, and P62 [23]). Compared with those in HCT116 cells, the expression levels of ATG5, ATG7, and LC3B in HCT116-NKD1 cells were dramatically decreased, whereas the P62 protein level in HCT116 cells was markedly increased relative to that in HCT116 cells (Fig. 5A), indicating that NKD1 inhibited the protein expression of the autophagy signaling pathway. We further validated whether NKD1 affects the formation of autophagosomes in cells via transmission electron microscopy (TEM). The results revealed that the number of autophagosomes in HCT116-NKD1 cells was significantly lower than that in HCT116 cells (Fig. 5B), demonstrating that NKD1 could suppress the production of autophagosomes in colon cancer cells. SW620 cells and SW620-nkd1^−/−^ cells were also assessed by Western blotting and TEM assays. Compared with those in SW620 cells, the expression levels of ATG5, ATG7, and LC3B were markedly increased in SW620-nkd1^−/−^ cells, whereas the P62 protein level was notably decreased in SW620-nkd1^−/−^ cells compared with that in SW620 cells (Fig. 5C), demonstrating that NKD1 gene knockout significantly enhanced the autophagy signaling pathway. TEM analysis further confirmed that NKD1 knockout obviously increased the number of autophagosomes in SW620-nkd1^−/−^ cells relative to SW620 cells (Fig. 5D). In summary, NKD1 inhibits the autophagy signaling pathway in colon cancer cells.

### NKD1 promotes MYC protein nuclear translocation and the expression of MYC downstream target genes

**Fig. 6:**
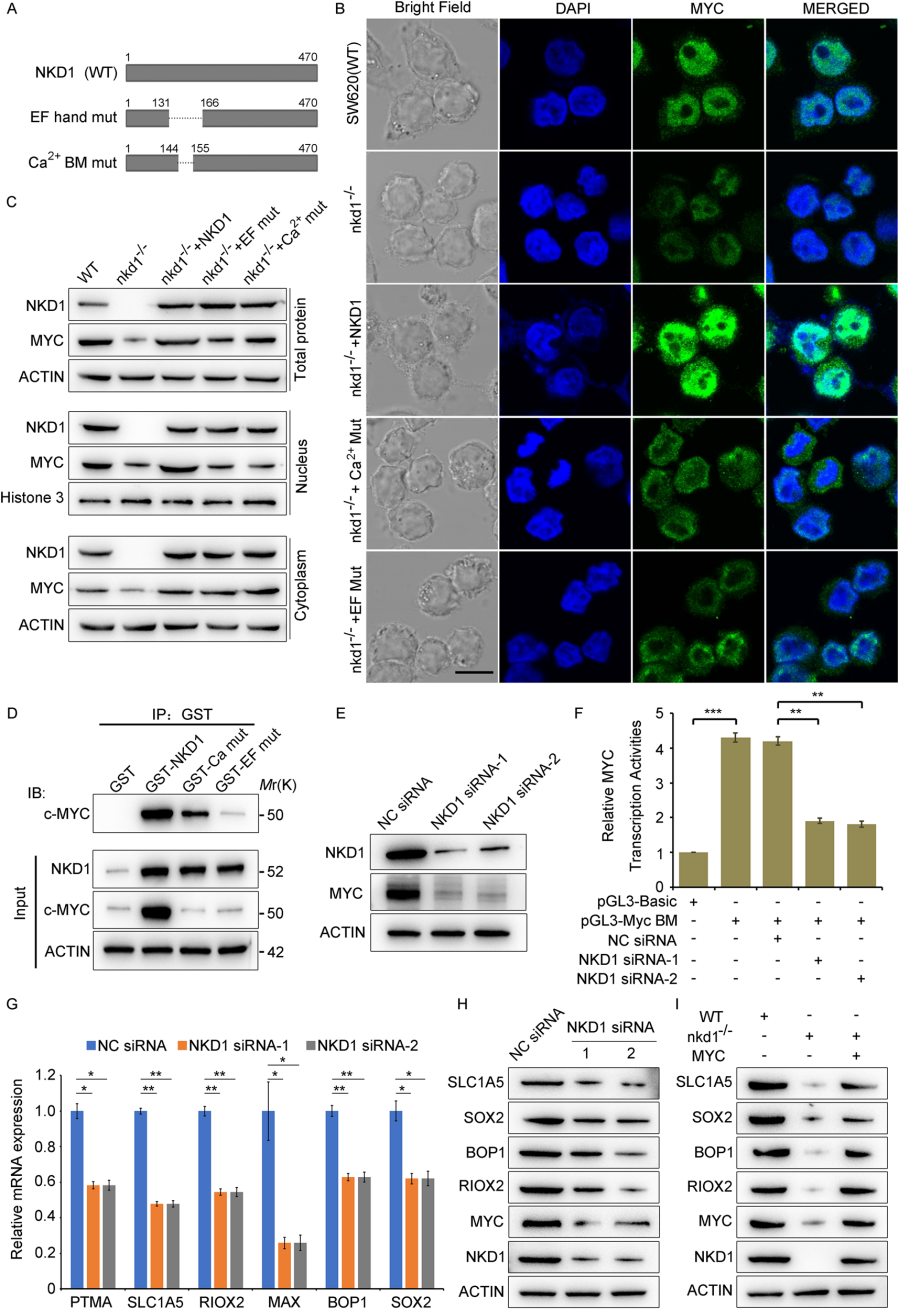
NKD1 advances MYC protein nuclear translocation and administer the expression of MYC downstream target genes. **A** Pattern diagram of NKD1 protein (WT), EF-hand deletion mutation and Ca^2+^ ion-binding motif (BM) deletion mutation. **B** Immunofluorescence detection of MYC protein nuclear localization in SW620, SW620-nkd1^−/−^, and SW620-nkd1^−/−^ cells transfected with pCMV-NKD1 plasmids (500 ng), pCMV-NKD1-EF-mut plasmid (500 ng), or pCMV-NKD1-Ca-mut plasmids (500 ng). The scale bar represents 50 μm. **C** Protein expression levels of MYC and NKD1 gene in the nucleus and cytoplasm in SW620, SW620-nkd1^−/−^, and SW620-nkd1^−/−^ cells transfected with pCMV-NKD1 plasmid (500 ng), pCMV-NKD1-EF-mut plasmid (500 ng), or pCMV-NKD1-Ca-mut plasmid (500 ng), were determined by western blotting. **D** The binding ability between the MYC and full-length NKD1, NKD1 Ca^2+^-binding motif deletion mutant, and NKD1 EF-hand deletion mutant protein were detected by immunoprecipitation experiments. HCT116 cells were transfected with pCMV-GST-Neo plasmids (500 ng), pCMV-GST-NKD1 plasmids (500 ng), pCMV-GST-NKD1-EF hand-deletion-Neo plasmids (500 ng), pCMV-GST-NKD1-Calcium ion domain deletion mutant plasmids (500 ng), respectively, after 48 h, collected the cells, gently lysed and added equal volumes of GST magnetic beads to the different supernatants, the next procedures was according to the immunoprecipitation methods to prepare protein samples, and the same volumes of different protein samples were used for western blotting. **E** The protein levels of NKD1 and MYC in SW620 cells transfected with NC siRNA (100 nM), NKD1 siRNA-1 (100 nM), and NKD1 siRNA-2 (100 nM) were detected by western blotting. **F** Relative MYC transcription activity were measured by Double-luciferase reporter gene experiments in SW620 cells transfected with pRL-TK plasmids (50 ng), pGL3-Basic plasmid (100 ng), pGL3-3XMYC-Bind Motif (BM) (100 ng), NC siRNA (100 nM), NKD1 siRNA-1 (100 nM) or NKD1 siRNA-2 (100 nM), as indicated. **G** The mRNA and protein (**H**) expression levels of downstream target genes of MYC protein were detected by Real time quantitative PCR and Western blotting in SW620 cells transfected with NC siRNA (100 nM), NKD1 siRNA-1 (100 nM) or NKD1 siRNA-2 (100 nM), respectively. **I** The protein expression levels of downstream target genes of MYC were detected by western blotting in parental SW620, SW620-nkd1^−/−^, and SW620-nkd1^−/−^ cells transfected with pLV3-MYC plasmid (500 ng). Each experiments was repeated at least three times, *p <0.05, **p <0.01, ***p <0.001.

To gain a deeper understanding of the biological function of the NKD1 protein, we analyzed its amino acid sequence and discovered that the NKD1 protein contains an EF-hand domain with a calcium ion-binding domain in the middle (Fig. 6A). We then constructed EF-hand deletion mutant plasmids (pCMV-NKD1-EF-mut) and calcium binding domain deletion mutant plasmids (pCMV-NKD1-Ca-mut). SW620 cells, SW620-nkd1^−/−^ cells, and SW620-nkd1^−/−^ cells transfected with pCMV-NKD1, pCMV-NKD1-EF-mut, or pCMV-NKD1-Ca-mut plasmids, respectively, were used to detect the effect of NKD1 on the localization of the MYC protein in the nucleus through laser confocal experiments. The images revealed that the signal intensity of the MYC protein in the nucleus in SW620-nkd1^−/−^ cells was obviously lower than that in SW620 cells. Transfection of the pCMV-NKD1 plasmid in SW620-nkd1^−/−^ cells substantially increased nuclear MYC content, whereas this increase was slight after pCMV-NKD1-Ca-mut plasmid transfection, with more MYC proteins distributed on the cell nuclear membrane. However, transfection of the pCMV-NKD1-EF-mut plasmid in SW620-nkd1^−/−^ cells did not result in obvious nuclear MYC protein changes (Fig. 6B), suggesting that the EF-hand domain of the NKD1 protein facilitates MYC protein nuclear entry. Furthermore, detection of MYC protein levels in the cytoplasm and nucleus via western blotting demonstrated that the protein expression levels of MYC in SW620-nkd1^−/−^ cells were notably lower than those in SW620 cells. Additionally, the transfection of NKD1 in SW620-nkd1^−/−^ cells significantly increased the expression level of the MYC protein, whereas the transfection of the pCMV-NKD1-EF-mut plasmid or pCMV-NKD1-Ca-mut plasmid obviously increased the cytoplasmic levels but not nuclear MYC levels (Fig. 6C). These results demonstrate that the EF-hand domain of the NKD1 protein promotes MYC protein nuclear entry. Furthermore, immunoprecipitation assays revealed that the NKD1 protein mutant without the calcium ion binding domain (GST-Ca mut) demonstrated obviously reduced MYC protein binding capabilities relative to that of the NKD1 wild-type protein (GST-NKD1). The EF-hand mutant protein (GST-EF mut) could hardly bind to MYC, indicating that the EF-hand domain of NKD1 is the key domain for NKD1-MYC protein interactions (Fig. 6D). Next, we investigated whether NKD1 could regulate the expression of MYC downstream target genes. We subsequently cloned three connected MYC-binding motif sequences (5’-CACGTG-3’) into pGL3-Basic plasmids (pGL3-3xMYC binding motif), and designed two small interfering RNA fragments that effectively interfered with the NKD1 gene, revealed by western blotting (Fig. 6E). Dual-luciferase reporter gene assays revealed that knocking down NKD1 downregulated nearly half of MYC’s transcriptional activities (Fig. 6F), suggesting that NKD1 could inhibit the expression of downstream target genes through MYC. To further verify the regulatory effect of NKD1 on downstream target genes of MYC, SW620 colon cancer cells were transfected with NC siRNA, NKD1 siRNA-1, or NKD1 siRNA-2, and several MYC downstream target genes, such as PTMA [24], SLC1A5 [25], RIOX2 [26], MAX, BOP1 [27], and SOX2 [28] on the basis of previously reported methods and analyzed by qRT-PCR and western blotting. These results further verified that NKD1 increases the expression of these MYC downstream target genes (Fig. 6G, H). In addition, SW620 cells, SW620-nkd1^−/−^ cells transfected with pLV3 control plasmids, or pLV3-MYC plasmids were used to further determine the effect of NKD1 on the expression of MYC downstream target genes. Western blotting indicated that the expression levels of downstream target genes of MYC in SW620-nkd1^−/−^ cells were extraordinarily lower than those in SW620 cells, whereas the expression levels of downstream target genes of MYC were notably increased in SW620-nkd1^−/−^ cells transfected with pLV3-MYC plasmids (Fig. 6I), indicating that NKD1 could increase the expression levels of MYC downstream target genes through MYC. In summary, the EF-hand domain in the NKD1 protein is the key component that promotes the entry of MYC into the nucleus and binds to the MYC protein; NKD1 can increase the expression of MYC downstream target genes through MYC.

### The EF hand domain of NKD1 is a key component in obstructing the apoptosis of colon cancer cells

**Fig. 7:**
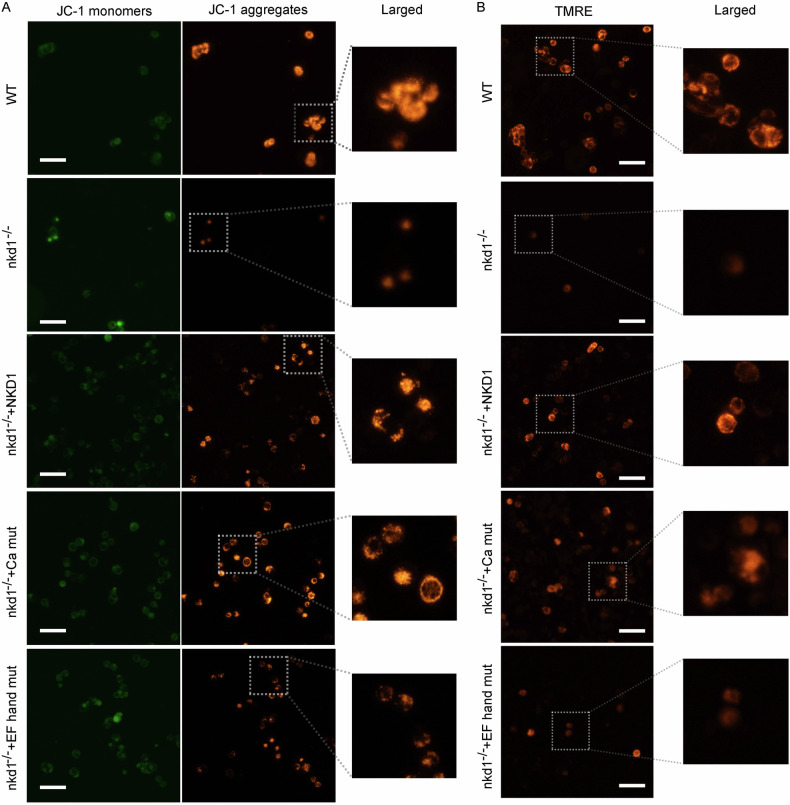
EF hand is the key domain of NKD1 in repressing apoptosis of colon cancer cells. **A** The apoptosis of parental SW620 cells, SW620-nkd1^−/−^ cells transfected with pcDNA3.1 plasmids (500 ng), or SW620-nkd1^−/−^ cells transfected with pcDNA3.1-NKD1 plasmids (500 ng), pcDNA3.1-NKD1 Ca^2+^-binding motif deletion mutant plasmids (500 ng) or pcDNA3.1-NKD1 EF-hand deletion mutant plasmids (500 ng), respectively, were measured by JC-1 staining and TMRE experiments (**B**).

Our previous studies revealed that NKD1 promotes the proliferation of colon cancer cells; however, the effect of NKD1 on cell apoptosis has not yet been reported. SW620 cells, SW620-nkd1^−/−^ cells, and SW620-nkd1^−/−^ cells transfected with pCMV-NKD1, pCMV-NKD1-EF-mut, or pCMV-NKD1-Ca-mut plasmids were used to perform JC-1 fluorescent probe experiments, and the results suggested that JC-1 monomers were significantly increased and that aggregates were obviously reduced in SW620-nkd1^−/−^ cells compared with SW620 cells, indicating that NKD1 gene knockout promotes cell apoptosis. Transfection of the NKD1 gene or the calcium deficient mutant gene in SW620-nkd1^−/−^ cells significantly increased the number of JC-1 aggregates, whereas transfection of the EF-hand deficient mutant gene only slightly increased the number of JC-1 aggregates (Fig. 7A), suggesting that the EF-hand domain is the key component of NKD1 protein in the inhibition of colon cancer cell apoptosis. Moreover, TMRE (a fluorescent dye that selectively labels active mitochondria) experiments were also conducted, and the number of TMRE-stained SW620-nkd1^−/−^ cells was significantly lower than that of SW620 cells, indicating that NKD1 represses the cell apoptosis. Transfection of the NKD1 gene into SW620-nkd1^−/−^ cells notably increased the number of TMRE-stained cells. Transfection of calcium deficient mutant genes partially enhanced the staining ability of TMRE, whereas transfection of the EF-hand deficient genes did not notably increase the staining ability of TMRE (Fig. 7B), confirming that the EF-hand domain of the NKD1 protein is key in inhibiting cell apoptosis. In brief, the EF-hand domain is a key component of NKD1 in blocking the apoptosis of colon cancer cells.

### NKD1 promotes proliferation, migration, and angiogenesis of colon cancer cells through MYC

**Fig. 8:**
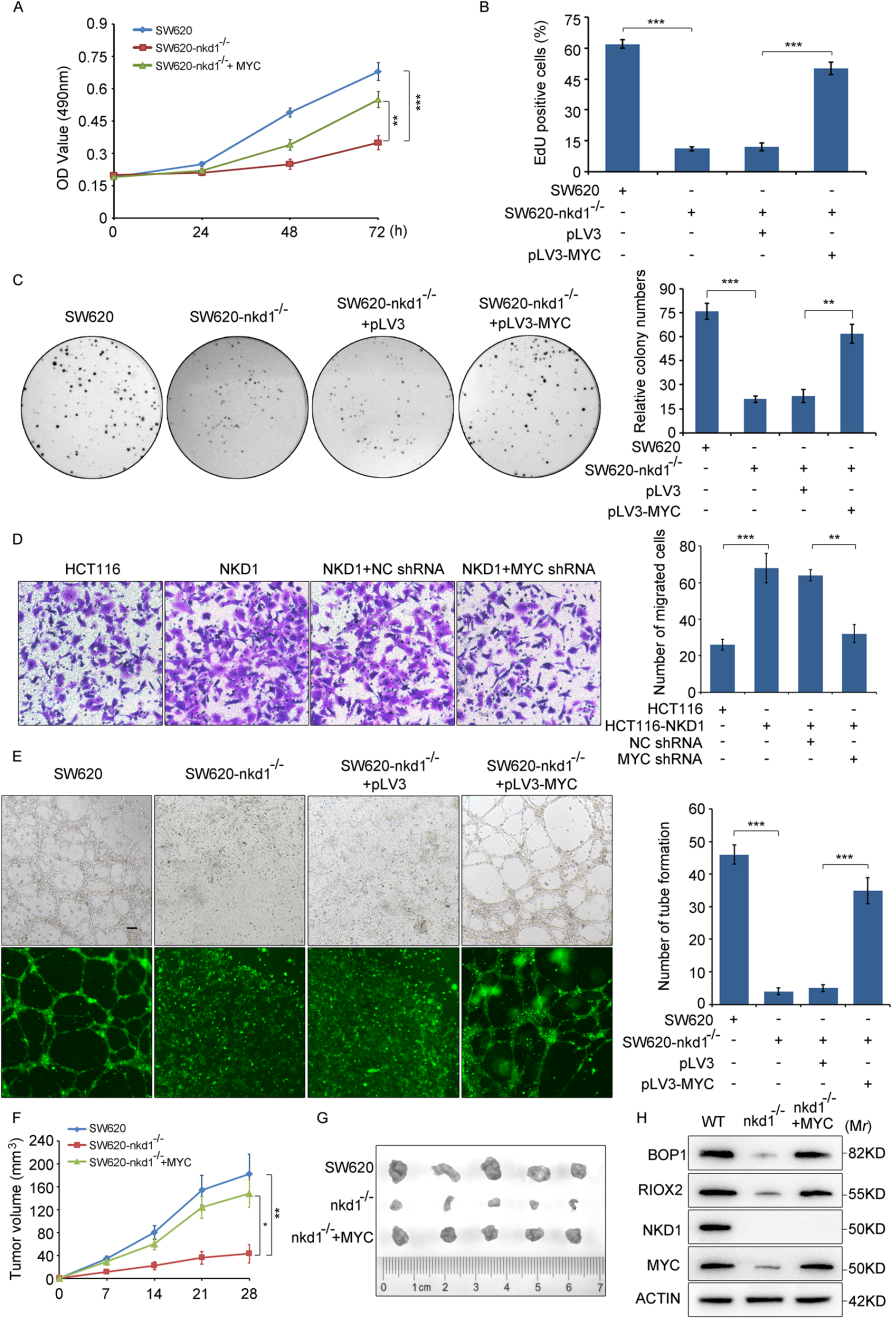
NKD1 upgrades colon cancer cell proliferation, migration, and angiogenesis in *vitro* and in *vivo* experiments through MYC. **A** The proliferation of parental SW620, SW620-nkd1^−/−^, and SW620-nkd1^−/−^ cells transfected with pLV3-MYC plasmid (500 ng) were measured by MTT assays. **B** Cell proliferation of SW620, SW620-nkd1^−/−^, and SW620-nkd1^−/−^ cells transfected with pLV3 plasmid (500 ng) or pLV3-MYC plasmid (500 ng) were determined by EdU and colony formation assays (**C**), on the right was the quantification of the number of colonies. **D** The migration of parental HCT116, HCT116-NKD1, and HCT116-NKD1 cells transfected with pLV3 plasmid (500 ng), or pLV3-MYC-shRNA plasmid (500 ng) was detected by Transwell experiments. On the right was a quantitative analysis of the number of migrating cells. **E** The effect of culture media of SW620, SW620-nkd1^−/−^, and SW620-nkd1^−/−^ cells transfected respectively with pLV3 plasmid (500 ng) or pLV3-MYC plasmid (500 ng) on the angiogenesis of the HUVEC cells was measured by the angiogenesis experiments. On the right was the quantity statistics of the blood vessels formed by HUVEC cells. **F** Tumor volume of nude mice with subcutaneous injection of SW620, SW620-nkd1^−/−^-pLV3 and SW620-nkd1^−/−^-pLV3-MYC cells were measured at different days. **G** The tumor tissues isolated from exnograft nude mice with subcutaneous injection of SW620, SW620-nkd1^−/−^-pLV3 and SW620-nkd1^−/−^-pLV3-MYC cells. **H** Protein expression levels of BOP1, RIOX2, NKD1, and MYC from the tumor tissues of nude mice were detected by western blotting.

We previously confirmed that NKD1 increases MYC protein expression. Because MYC is a widely researched oncoprotein, we investigated whether NKD1 stimulates colon cancer progression through MYC. The SW620 cells, SW620-nkd1^−/−^ cells, and SW620-nkd1^−/−^ cells transfected with pLV3-MYC plasmids (SW620-nkd1^−/−^+MYC cells) were used to conduct MTT, EdU and colony formation experiments. These results demonstrated that the proliferation ability of SW620-nkd1^−/−^ cells was markedly lower than that of SW620 cells, whereas the proliferation ability of SW620-nkd1^−/−^+MYC cells was notably stronger than that of SW620-nkd1^−/−^ cells (Fig. 8A–C). Thus, knocking out the NKD1 gene in colon cancer cells exceptionally inhibited cell proliferation, and this inhibition could be eliminated by overexpression of MYC, indicating that NKD1 promotes cell proliferation through MYC. Additionally, HCT116 cells, HCT116-NKD1 cells, and HCT116-NKD1 cells transfected with pLV3-U6-Puro plasmids or pLV3-U6-MYC-shRNA-Puro plasmids, were used to determine the effect of NKD1/MYC signaling on cell migration ability. The Transwell assay results revealed that the migration ability of HCT116-NKD1 cells was markedly greater than that of HCT116 cells, whereas the transfection of pLV3-U6-MYC-shRNA-Puro plasmids into HCT116-NKD1 cells significantly reduced their migration ability relative to that of cells transfected with pLV3-U6-Puro plasmids (negative control siRNA, NC siRNA) (Fig. 8D). These findings demonstrate that NKD1 promotes colon cancer cell migration through MYC. Furthermore, SW620 cells, SW620-nkd1^−/−^ cells, and SW620-nkd1^−/−^ cells transiently transfected with pLV3 plasmids or pLV3-MYC plasmids, were used to determine the effect of NKD1/MYC signaling on angiogenesis. Compared with SW620 cells, SW620-nkd1^−/−^ cells obviously reduced the number of blood vessels formed by HUVECs, while transfecting SW620-nkd1^−/−^ cells with pLV3-MYC plasmids significantly increased the angiogenesis of HUVECs (Fig. 8E). These findings indicate that NKD1 increases the angiogenesis in colon cancer cells *via* MYC.

In addition to in vitro experiments, we conducted xenograft tumor model experiments in nude mice. We first constructed a stable SW620-nkd1^−/−^ cell line overexpressing the MYC protein (SW620-nkd1^−/−^+MYC cells). SW620 cells, SW620-nkd1^−/−^ cells and SW620-nkd1^−/−^+MYC cells were subsequently used to construct a xenograft tumor model via subcutaneous injection into the right hind legs of nude mice. We measured the volume of subcutaneous tumors in the nude mice weekly, revealing that the growth ability of SW620-nkd1^−/−^ cells was distinctly lower than that of SW620 cells, in these mice, whereas the proliferation ability of SW620-nkd1^−/−^+MYC cells was notably greater than that of SW620-nkd1^−/−^ cells (Fig. 8F). These findings indicate that NKD1 enhances the tumorigenesis of colon cancer cells in *vivo* through MYC. We removed and photographed the subcutaneous tumors from nude mice and found that knocking out the NKD1 gene resulted in a significant reduction in tumor volume, whereas overexpression of MYC in SW620-nkd1^−/−^ cells markedly increased the tumor volume (Fig. 8G). These data further demonstrate that NKD1 promotes the tumor growth through MYC in *vivo*. Additionally, we extracted tumor tissues for western blot detection of the expression of downstream target genes of MYC, revealing that NKD1 gene knockout could decrease BOP1 and RIOX2 protein expression. Conversely, overexpressing MYC in SW620-nkd1^−/−^ cells markedly increased BOP1 and RIOX2 expression (Fig. 8H). These findings indicate that NKD1 upregulates BOP1 and RIOX2 protein expression through MYC. Taken together, these findings indicate that the NKD1/MYC signaling pathway promotes the proliferation, migration, and angiogenesis of colon cancer cells.

## Discussion

*Drosophila* and mouse Nkd proteins function as suppressors of the Wnt/β-catenin pathway [2, 29]. Researchers have reported that NKD1 expression is low in breast cancer[6], osteosarcoma [30], acute myeloid leukemia [4], hepatocellular carcinoma [5], and non-small-cell lung cancer tissues [31]. However, studies have also demonstrated that NKD1 is highly expressed in colorectal carcinoma tissues [7, 32]. Furthermore, this study also revealed that NKD1 could promote the colon cancer progression. These results strongly indicate that NKD1 is a tissue-specific biomarker for colon cancer. In studying the correlation between NKD1 expression and the WNT signaling pathway, we found that NKD1 increased of β-catenin protein expression in colon cancer cells with high NKD1 expression. Therefore, the expression of NKD1 in colon cancer cells is significantly positively correlated with the expression of the β-catenin gene in the classical Wnt pathway. However, the protein expression level of NKD1 is low in gastric and liver cancer cells, and the expression of NKD1 inhibits the expression of β-catenin. Therefore, in gastric and liver cancer cells with low NKD1 expression, β-catenin gene expression is significantly negatively correlated with that of NKD1.

In this study, we discovered that PPARδ is a transcription factor of the NKD1 gene. PPARδ is a member of the PPAR family, which contains PPARα, PPARδ, and PPARγ. PPARs are ligand-activated transcription factors that control the expression of multiple target genes involved in lipid metabolism, inflammatory responses, and tumor progression [33]. PPARδ has completely opposite functions to those of PPARα and PPARγ. Studies have demonstrated that PPARδ can promote colon cancer proliferation and migration [34, 35], whereas PPARα and PPARγ retard colon cancer progression [36, 37]. In addition, in exploring NKD1 gene transcription factors, we analyzed the promoter sequence of the NKD1 gene via an online website and identified dozens of potential transcription factor binding motifs. We selected only the top four potential transcription factors for verification and discovered PPARδ. However, this finding does not rule out the possibility that other transcription factors might also regulate the transcription of the NKD1 gene. We will also screen for other potential transcription factors in future studies to elucidate the upstream regulatory mechanism of the NKD1 gene.

To date, studies have revealed that the degradation of the MYC protein occurs mainly through the proteasome ubiquitination pathway [18, 38–40]; however, the degradation mode of the MYC protein in colon cancer has not yet been reported. We found that the MYC protein was degraded mainly through the autophagy pathway. However, when the proteasome degradation pathway was blocked, the MYC protein accumulated over time (Supplementary Fig. SF2). In contrast, inhibition of the autophagic degradation pathway led to a faster accumulation rate of the MYC protein (Fig. 4C). These results indicated that the MYC protein was degraded mainly through the autophagy pathway in colon cancer cells, althrough a small portion of the protein was also degraded through the proteasome pathway. In addition, NKD1 also suppressed the interaction between the MYC and LC3B proteins. Therefore, NKD1 maintains the high expression of MYC protein in cells by inhibiting MYC protein autophagic degradation. Interestingly, we further discovered that NKD1 could inhibit the autophagy signaling pathway, and TEM analysis confirmed this finding (Fig. 5). Overall, the PPARδ/NKD1/MYC signaling pathway promotes the colon cancer progression (Supplementary Fig. SF3), and NKD1 could serve as a specific biomarker and corresponding drug target for colon cancer.

## Materials and methods

### Construction of plasmids

The dual luciferase reporter plasmids were constructed by inserting the NKD1 promoter region (-1826 to -429 bp; -1626 to -429 bp; -1426 to -429 bp; -1226 to -429 bp; -1026 to -429 bp; -626 to -429 bp) into the pGL3-Basic plasmids to generate the pGL3-1398, pGL3-1198, pGL3-998, pGL3-798, pGL3-598, and pGL3-198 plasmids, respectively, as previously described [41]. The pGL3-598 plasmids were used to construct the SP1-binding motif mutant, FOXC2-binding motif mutant, PPARδ-binding motif mutant and the CTCFL-binding motif mutant plasmids. The pLV3-CMV-MYC-3XFLAG-Puro plasmids (pLV3-MYC plasmids), pCMV-GST-NKD1-Neo plasmids, pCMV-GST-NKD1-Calcium ion domain deletion mutant plasmids, pCMV-GST-NKD1-EF hand deletion mutant plasmids, pLV3-U6-Puro plasmids, pLV3-U6-MYC-shRNA-Puro plasmids, and pcDNA3.1-PPARδ were all purchased from the Miaoling Biology (WuHan City, China).

### Dual luciferase reporter experiments

The constructed pGL3-NKD1 promoter plasmids (200 ng) and the pGL3-598 mutant plasmids (200 ng) were transfected into colon cancer HCT116 cells as previously described [42].

### Colon cancer cell culture and transfection

In this study, colon cancer HCT116 cells, SW480 cells, HT29 cells, SW620 cells, and HCT116-NKD1 cells stably overexpressing NKD1 were constructed by transfecting a lentiviral vector into parental HCT116 cells, and SW620-nkd1^−/−^ cells were successfully generated previously with nkd1 gene knockout in parental SW620 cells [7]. Colon cancer cells were cultured according to previous methods [41]. Cancer cells were transfected with Lipo2000 transfection reagent according to the manufacturer’s instructions. The two NKD1 small interfering fragment sequences used in the experiments are: NKD1 siRNA-1: CCAGAAGGCUCAUGGGAAATT; NKD1 siRNA-2: ACAGAAACUUGGUGGGAAATT.

### Western blotting assays

The detailed procedures were as previously described [43]. The dilution ratios of the different antibodies used were as follows: BOP1 (abmart PU147657) 1:1000, c-MYC (Abcam ab32072) 1:1000, RIOX2 (Sangon Biotech D225067) 1:800, SLC1A5 (Proteintech 20350-1-AP) 1:7000, MAX (Proteintech 10426-1-AP) 1:600, SOX2 (Proteintech 11064-1-AP) 1:600, and NKD1 (QYAOBIO) 1:1000.

### Differential protein expression profiling analysis of SW620 cells and SW620-nkd1^−/−^ cells

The differential protein expression of SW620 cells and SW620-nkd1^−/−^ cells were performed with isobaric tags for relative and absolute quantitation technology by Sangon Biotech Company. Differential proteins were plotted as volcanoes after adjusting P-values by FDR control.

### Bioinformatics analysis

The mRNA expression levels of the NKD1 and MYC genes downloaded from TCGA database (https://xenabrowser.net/datapages/) and GEO datasets (GSE17536 and GSE29621), saved in Supplementary Table 4, which were analyzed via the Sangerbox 3.0 network software (http://www.sangerbox.com/home.html).

### Immunohistochemistry experiments

The immunohistochemistry assays were performed according to previous methods [44], and the antibody dilution ratios used were as follows: NKD1 (QYAOBIO, 1:200) and c-MYC (Abcam, 1:200).

### Immunofluorescence and laser confocal microscopy

Most experiments were performed as previously described [7, 44], and the antibody dilution ratios used were as follows: NKD1 (QYAOBIO, 1:200), c-MYC (Abcam, 1:200), and LC3B (Proteintech, 1:200); the scale bar represents 50 μm.

### Immunoprecipitation assays

The detailed procedures were perfromed according to previous methods [7].

### Quantitative real-time PCR (qRT-PCR)

Total mRNA was isolated from colon cancer HCT116 cells, SW480 cells, parental SW620 cells, SW620-nkd1^−/−^ cells, and SW620-nkd1^−/−^ cells transfected with pLV3-MYC plasmids according to the manufacturer’s instructions, and other procedures were the same as previously described [44]. The primers used in this study are listed in Supplementary Table 1.

### Tumor proliferation experiments

The MTT, EdU, and colony formation assays were performed as previously described [7].

### Transwell assays

Transwell cell preparation: Briefly, 100 µl of diluted Matrigel (3.9 μg/µl) was added to the upper chamber, which was then incubated at 37 °C for 30 min to polymerize the Matrigel into a gel. The chamber was placed into a 24-well culture plate, and 500 µl of preheated serum-containing medium was added to the lower chamber. Preparation of the cell suspension: the cells were digested and centrifuged, the culture medium was discarded after the digestion was terminated, the cells were washed with PBS 1–2 times, and the cells were resuspended in serum-free medium containing BSA. The cell density was adjusted to 3 × 10^5^. A total of 150 µl of the cell suspension was added to the Transwell chamber, and the cells were conventionally cultured for 36 h. The samples were fixed with 4% paraformaldehyde for 30 min, dyed with crystal violet for 15 min, then cleaned, air dried, and imaged.

### Angiogenesis experiments

HUVECs were cultured overnight to 80% confluence and subsequently collected and counted. Two hundred microliters (~120,000 cells) of each HUVEC suspension was added to a 24-well plate. After 4 h in a 37 °C incubator, the formation of blood vessels was observed. The culture medium (24 h) of the experimental group (parental SW620 cells, SW620-nkd1^−/−^ cells, and SW620-nkd1^−/−^ cells transfected with pLV3-MYC plasmids) was added, and images were collected for analysis 15 h later. For fluorescence staining, the culture medium in the upper well was carefully removed to avoid touching the glue or cell network; next, 50 μl of calcein diluted with serum-free culture medium was added to final concentration of 6.25 μg/ml. The mixture was incubated in a 37 °C incubator for 30 min in the dark, rehydrated once with PBS, and imaged.

### Transmission electron microscopy (TEM) analysis

Preparation of TEM samples: HCT116, HCT116-NKD1, SW620, and SW620-nkd1^−/−^ cells in a culture dish were slowly scraped using a scraper, and 50 µl of glutaraldehyde fixative was added and mixed well. The samples were then placed in a pointed bottom centrifuge tube and centrifuged at 4 °C and 1500 rpm for 8 min. The supernatant was carefully aspirated, 4% glutaraldehyde fixative was slowly added, the mixture was fixed, for 3–4 h, and then the cells along the centrifuge tube wall were gently removed with a cell pick needle to separate the cell clusters from the tube wall for better fixation. The cell clusters were cut into small clumps of 2 mm^3^ with a sharp blade, sealed in a centrifuge tube filled with 4% glutaraldehyde fixative, and mailed to the Zhenjiang Zhuanbo Testing Technology Co., Ltd. Then, postprocessing was performed on the cell blocks, and the fixed cell blocks were cleaned, fixed with osmium acid, washed, dehydrated, soaked in resin, and processed into embedding blocks. The samples were then repaired, ultrathin sliced, and metal-stained prior to observation, and imaging via TEM. Single intact cells were photographed at 3.0 K zoom; local areas were magnified to 10.0 K.

### Tumor formation in nude mice

SW620-nkd1^−/−^ cells were transfected with pLV3 control plasmids or pLV3-MYC plasmids for 2 days, after which the cells were screened with purinomycin for 2 weeks to generate SW620-nkd1^−/−^-pLV3 and SW620-nkd1^−/−^-MYC cells stably expressing MYC proteins. The parental SW620, SW620-nkd1^−/−^-pLV3, and SW620-nkd1^−/−^-MYC cells were subcutaneously injected into the right lower limb of 7-week-old female nude mice. After the injection, the tumorigenic volume was measured every 7 days, and the tumors were isolated 28 days later after euthanizing the mice.

### Statistics

All the experiments were conducted at least three times. Unpaired two-tailed Student’s *t* test methods were used to analyze the independent experimental groups. The differences considered significant were as follows: *p < 0.05; **p < 0.01; and ***p < 0.001.

## Supplementary information

Supplementary information

Supplementary Table ST1

Supplementary Table ST2

Supplementary Table ST3

Supplementary Table ST4

Original Figure 1

Original Figure 2

Original Figure 3

## Data Availability

The data supporting the findings of this study are available from the corresponding author upon reasonable request.
