# Supplementary material for: NKD1 enhances colon cancer progression by inhibiting the autophagic degradation of MYC
Source: Cell Death Dis. 2025 Jul 17;16(1):532. doi: 10.1038/s41419-025-07875-8 (PMC12271375; doi:10.1038/s41419-025-07875-8)
Supplement: Supplementary file 1 — Supplementary information [file 41419_2025_7875_MOESM1_ESM.docx]

**
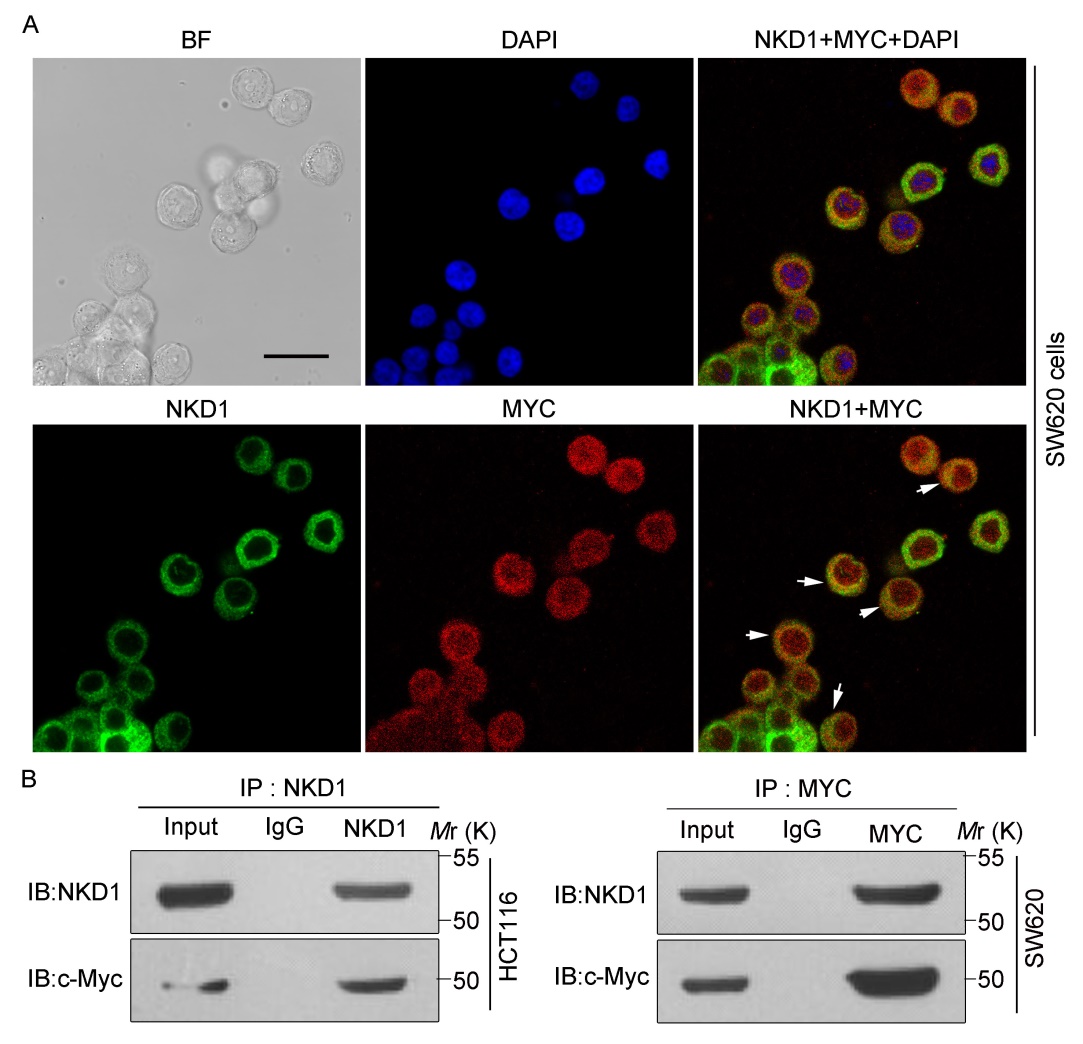
**

**Supplementary Figure SF1.**

A. Laser confocal microscopy experiment detected the localization of the NKD1 and MYC protein in colon cancer SW620 cells. B. Immunoprecipitation assays examined the binding ability of NKD1 and MYC proteins in colon cancer HCT116 and SW620 cells.

**
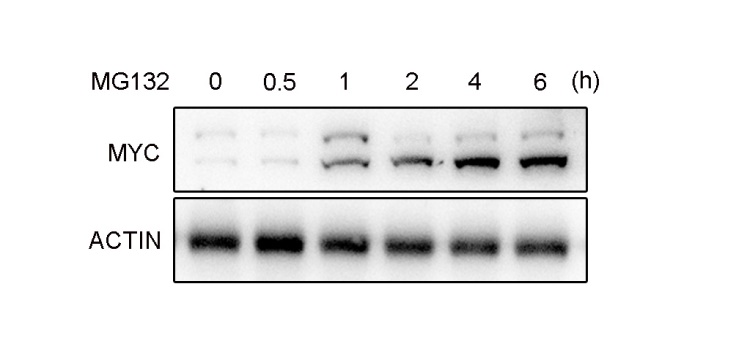
**

**Supplementary Figure SF2.**

The expression levels of MYC protein in colon cancer HCT116 cells treated with MG132 for 0.5, 1, 2, 4, 6 hours, respectively, were detected by western blotting.


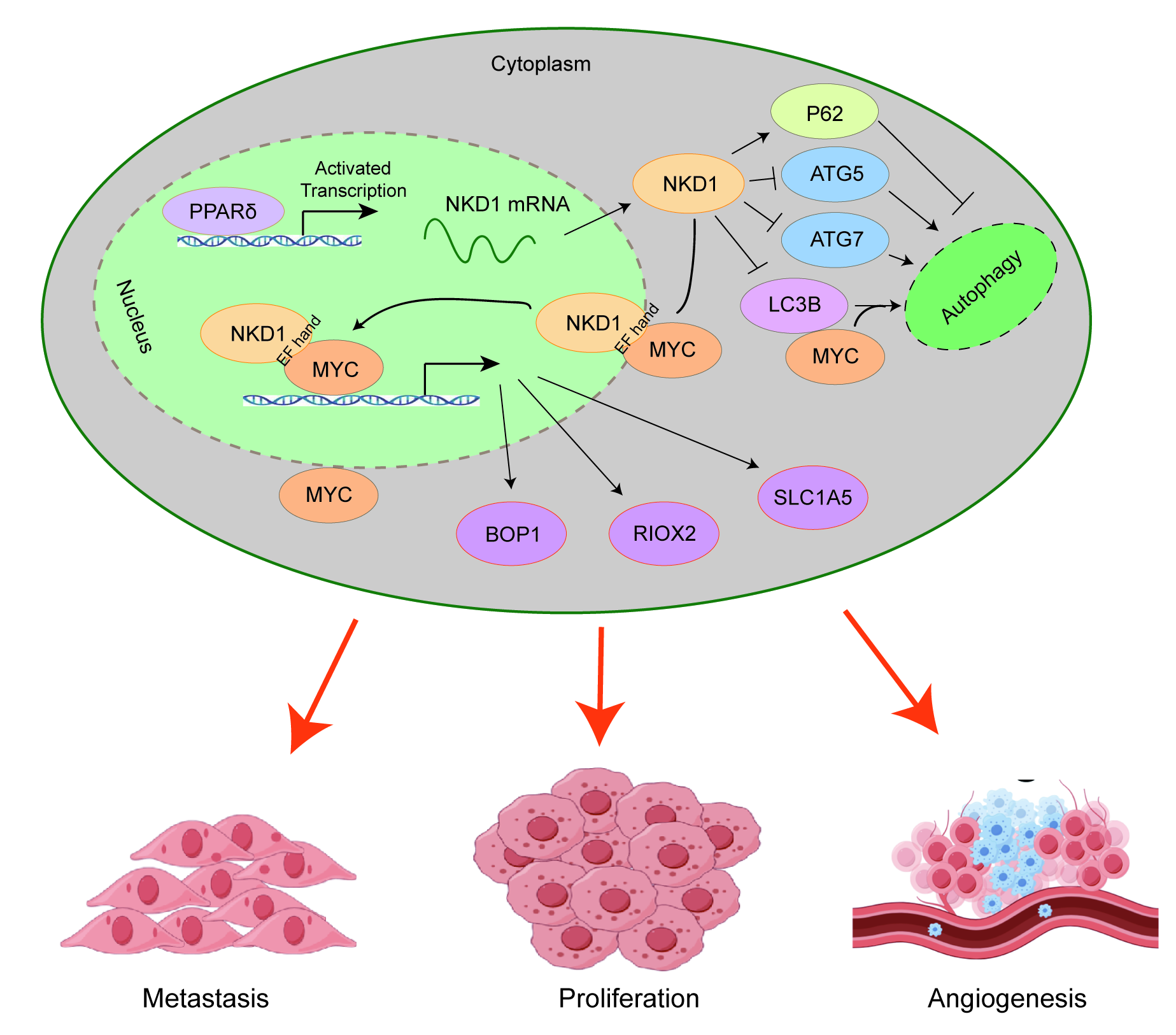


**Supplementary Figure SF3. The model diagram inferred from this study.**

PPARδ activated NKD1 gene transcriptional expression. NKD1 prohibited the MYC protein autophagic degradation by hampering the autophagy signaling pathway and preventing the interaction between the MYC and LC3B proteins, which maintains the high levels of MYC protein in the cells. Moreover, NKD1 benefites MYC protein nuclear translocation and enhances the transcriptional expression of downstream target genes (such as, BOP1, RIOX2, MAX, SLC1A5 genes) by binding to MYC with the EF-hand domain of NKD1 proteins. Overall, the PPARδ/NKD1/MYC signaling pathway advances colon cancer progression, indicating that NKD1 would be a potential therapeutic target of colon cancer therapy.
