# Supplementary figures and images for: NKD1 enhances colon cancer progression by inhibiting the autophagic degradation of MYC
Source: Cell Death Dis. 2025 Jul 17;16(1):532. doi: 10.1038/s41419-025-07875-8 (PMC12271375; doi:10.1038/s41419-025-07875-8)

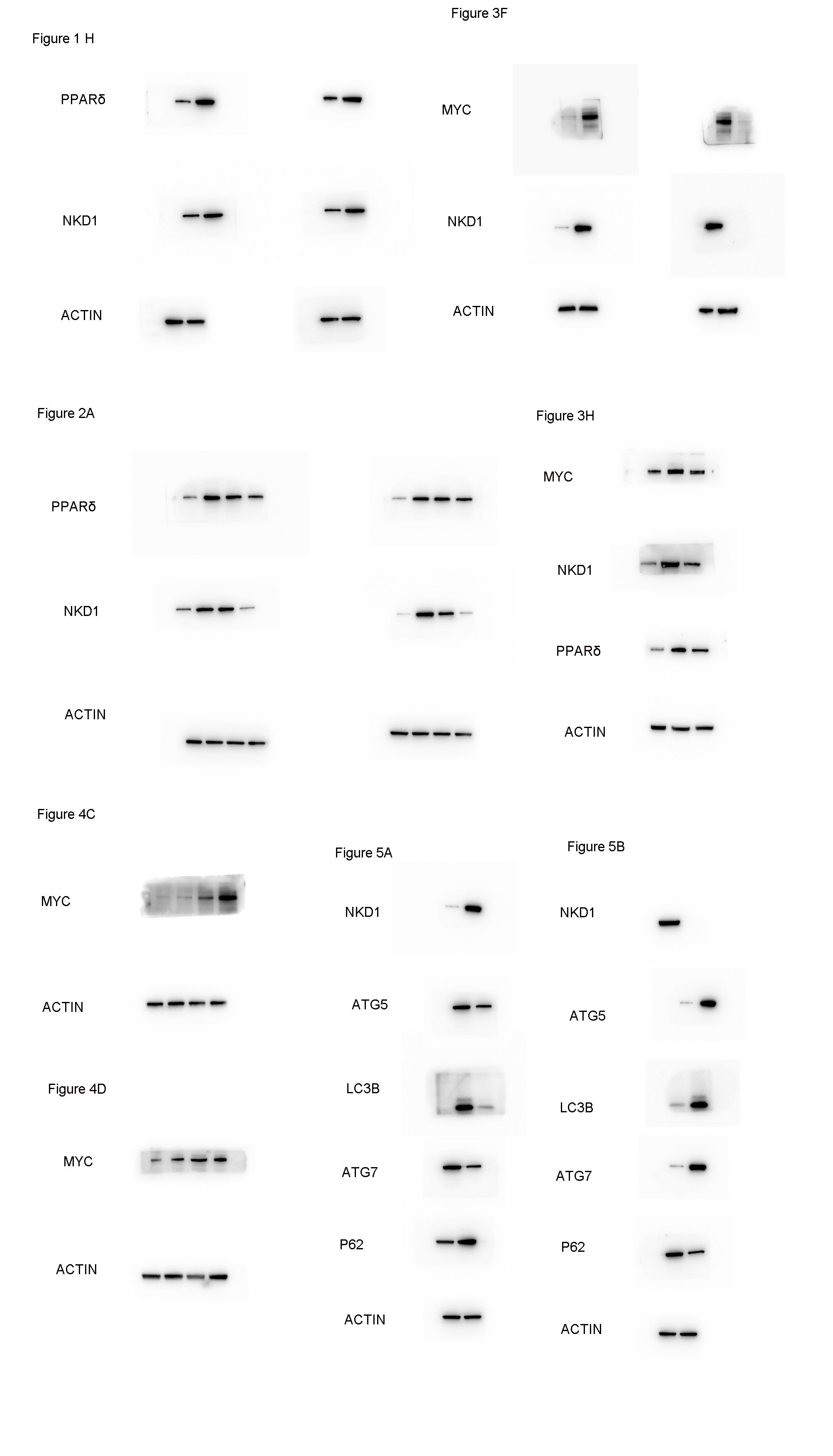

Supplement: Supplementary file 6 — Original Figure 1 [file 41419_2025_7875_MOESM6_ESM.tif]

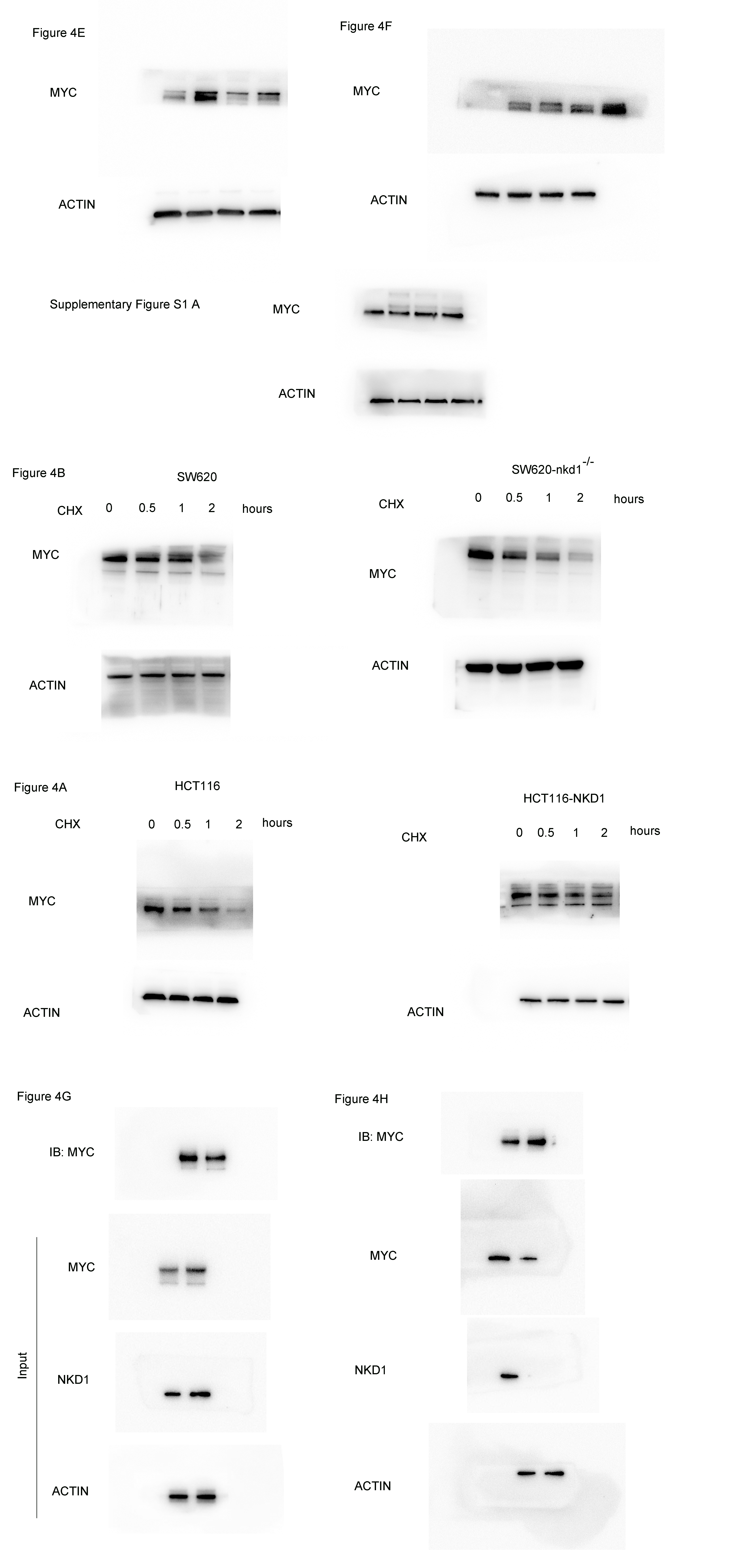

Supplement: Supplementary file 7 — Original Figure 2 [file 41419_2025_7875_MOESM7_ESM.tif]

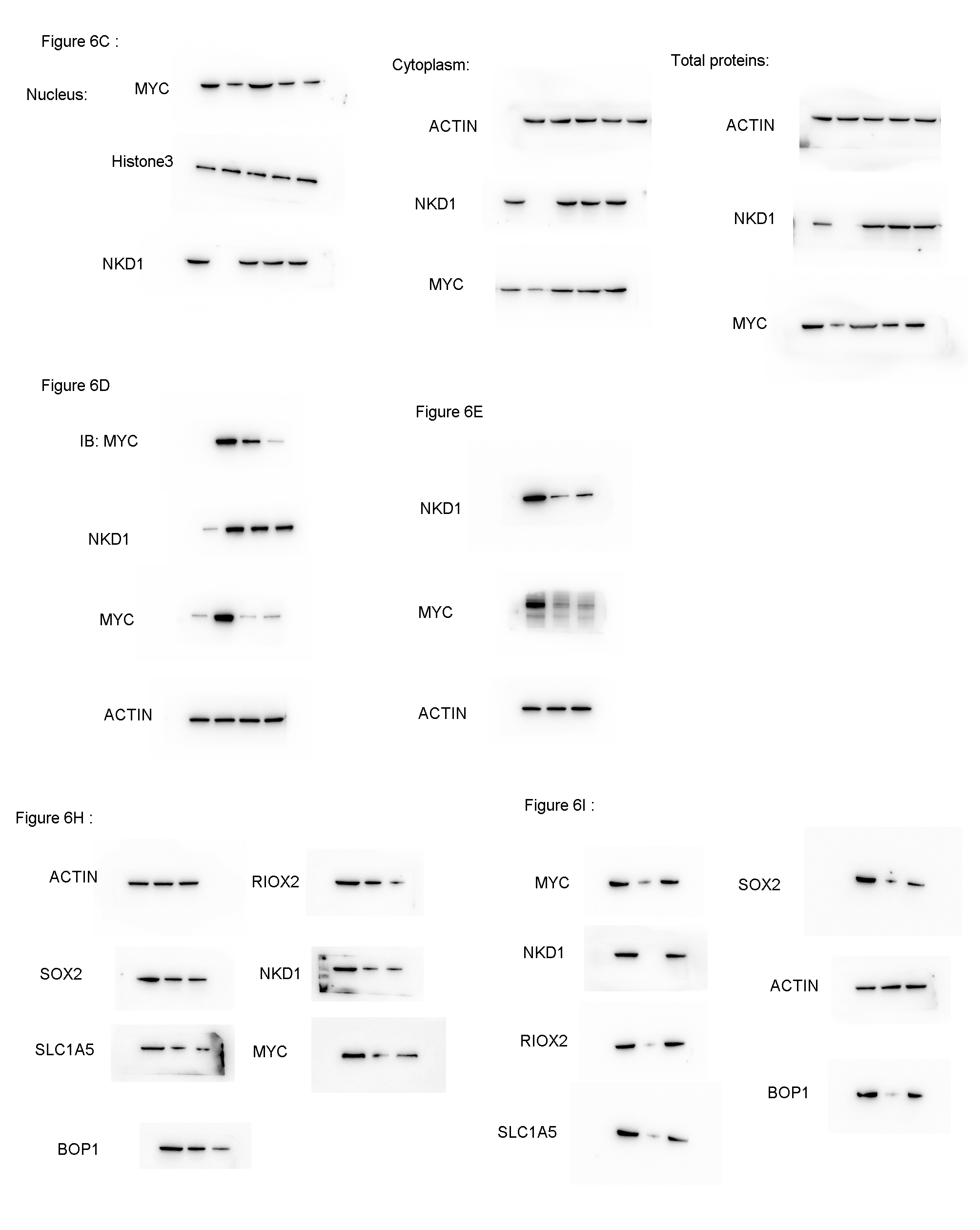

Supplement: Supplementary file 8 — Original Figure 3 [file 41419_2025_7875_MOESM8_ESM.tif]
